# VeloxChem Quantum–Classical Interoperability for Modeling of Complex Molecular Systems

**DOI:** 10.1021/acs.jpca.5c03187

**Published:** 2025-08-04

**Authors:** Juan Angel de Gracia Triviño, Iulia Emilia Brumboiu, David Carrasco-Busturia, Xin Li, Chenxi Li, Mathieu Linares, Valentin Lindfeld, Young Min Rhee, Julia Rune, Bastiaan van Hoorn, Patrick Norman, Mårten S. G. Ahlquist

**Affiliations:** † PDC Center for High Performance Computing, School of Electrical Engineering and Computer Science, 7655 KTH Royal Institute of Technology, SE-100 44 Stockholm, Sweden; ‡ Faculty of Physics, Astronomy and Informatics, 49577 Nicolaus Copernicus University in Toruń, 87-100 Toruń, Poland; § Division of Theoretical Chemistry and Biology, School of Engineering Sciences in Chemistry, Biotechnology and Health, 530019 KTH Royal Institute of Technology, SE-100 44 Stockholm, Sweden; ∥ Department of Chemistry, 34968 Korea Advanced Institute of Science and Technology (KAIST), Daejeon 34141, Korea

## Abstract

Being a program written primarily in Python that strictly adheres to modern object-oriented software engineering and parallel programming practices, VeloxChem is shown to be suitable for the development of (semi)­automatized workflows that extend its scope from first-principles quantum chemical purism to hybrid quantum–classical interoperability and some degree of semiempiricism. Methods are presented for building complex systems such as metal–organic frameworks, constructing molecular mechanics and interpolation mechanics force fields, conformer searches, system solvation, determining free energies of solvation, and determining free energy profiles of reaction pathways using the empirical valence bond method. The implementations are made intuitive with opportunities for interactive plotting and 3D molecular structure illustrations through the use of Jupyter notebooks.

## Graphical abstract

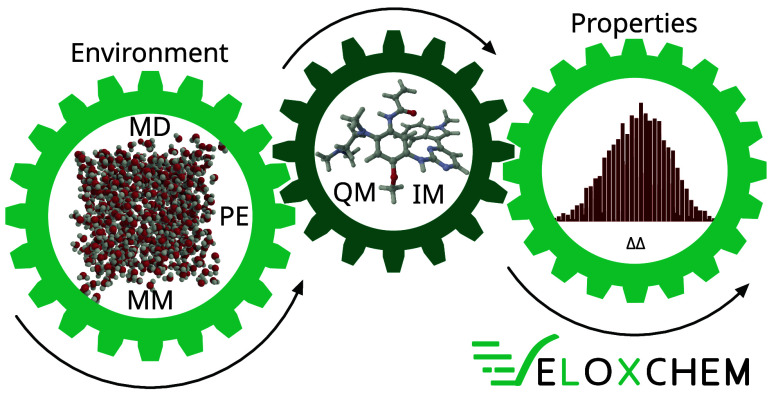

## Introduction

Physics-based models and simulation methods have become widely used to understand and interpret experimental observations, and they have become a standard tool also in the community of experimentalists. Today, quantum chemical methods, with density functional theory (DFT) as the most widely applied,^1^ are used in all fields of chemistry with applications ranging from the understanding of reaction energetics to light–matter interactions in spectroscopy. Challenges are still encountered, however, not least in studies of molecular systems in biochemistry and materials science, where there is a need to describe the often structurally complex environments, as exemplified by liquids, membranes, solid/liquid interfaces, and sites inside metal–organic frameworks. In fact, such complex systems often display significant thermal fluctuations and accessing the related diversity with simulation tools such as molecular dynamics (MD) becomes desirable.

Due to the large system size that would be required even for a minimal description of complex environments, very efficient computational methods are indispensable. In response to this need, methods based on classical physics such as molecular mechanics (MM) are very powerful in describing systems of larger sizes and can also propagate the systems over significant time scales. Using MD simulations based on MM, experimental observations related to phenomena dominated by intermolecular interactions can be accurately described, including diffusion,^2^ solvation free energies,^3^ and protein ligand interactions.^4,5^ A limiting factor of MM/MD often originates from the simplicity of analytic functions adopted in conventional MM force field models, which restricts their reliabilities especially when intramolecular interactions are important. In addition, MM models are, at least in practice, only applicable to electronic ground states, as the concept of MM atom types does not straightforwardly apply to excited states. Of course, refitting the force field parameters to excited state reference data is conceptually possible, but the same conventional analytic functions may not work as well for excited states. More importantly, extending MD to multistate dynamics often requires the state-to-state coupling information, whose fitting may become frustratingly cumbersome.

For this situation, a more general class of force fields based on the technique of interpolation mechanics (IM) is instead available.^6^ The IM approach builds a global potential from local harmonic expansions that in turn are determined at the level of quantum mechanics (QM).^7–9^ A ligand IM force field can be combined with protein and/or solvent MM force fields to conduct large-scale IM/MM MD simulations on the microsecond time scale. The accuracy in the ligand dynamics has the potential to equal that of the employed QM level and it is limited only by the efficacy of the expansion points.^10^ Because the harmonic expansions can be obtained with energy, gradient, and Hessian information, which with analytical/numerical approaches are routinely available from conventional QM methods, the IM approach does not rely on any predefined analytic functional form or numerical fitting. When state-to-state coupling information can be provided from the QM side, a multistate model^11,12^ can also be constructed. The obvious target aim with IM is to reach QM accuracy in the global potential with as few expansion points as possible and to automatize the entire procedure for building the potential.

From the perspective of computer programs, many well-developed codes exist that demonstrate extensive functionalities for both quantum chemistry calculations and classical MD simulations. However, a problem often arises when the system under study requires characterizing both its electronic structure properties such as bond breaking processes or electronic excitations, and its molecular structure progressions such as conformational adaptation of reaction intermediates^13–15^ or solvent responses to an electronic excitation.^16^ In such cases, multiscale modeling becomes necessary and, while it is possible to derive force field parameters for MD from quantum chemical calculations, either with MM or IM, it often entails data transfer between different sets of codes that are not seamlessly interoperable. This fact often results in long preparation times for simulations, and in our own experience, it sometimes even leads researchers to avoid certain aspects in their studies due to the labor-intensive and error-prone nature of the work. For condensed phase simulations of reaction intermediates or time-dependent DFT calculations of electronic transitions, the inclusion of solvation effects via an implicit model is a mere keyword away. Including explicit solvation effects using molecular dynamics, on the other hand, often requires learning a new code and manually transferring data between different code sets. In this regard, a seamless interoperability will greatly facilitate the whole process and make the construction of workflows far more straightforward. This will be the main focus of the present work. Herein, we will present functionalities of the VeloxChem software developed to facilitate the employment of hybrid simulation techniques that mix quantum chemistry and classical embedding toward performing molecular dynamics simulations. Its description will be centered around automatized force field generation as well as its related workflow implementations.

Our subsequent presentation is divided into two main sections. In addition to a brief description of the simple and straightforward software installation procedure, the first of these two describes the key software classes that allow the development of computational workflows. The second contains a selection of illustrative code snippet examples of the usage of the aforementioned classes. These code snippets are excerpts taken from complete and self-contained Jupyter notebooks that are provided in the Supporting Information.

## Software Structure and Installation

The VeloxChem software^17^ is open source distributed under the BSD License 2.0 and made available for installation from the Anaconda platform for the three main operating systems, namely Windows, macOS, and Linux. Presently, it is distributed via its own channel but with the planned release of version 1.0, it will instead adopt the more standard conda-forge channel. One can install the software needed for running all examples presented in this work with a single conda install command:

conda install veloxchem jupyterlab -c veloxchem -c conda-forge

The main text of this article presents code snippets that demonstrate some of the science enabling functionality and automatization features in VeloxChem with a focus on the QM and MM/MD boundary of simulations in theoretical chemistry. As mentioned, these snippets are excerpts from Jupyter notebooks provided in the Supporting Information, and for the execution of these notebooks, we recommend installing and using JupyterLab that is therefore also included in the conda install command.

The VeloxChem package has several dependencies that the conda package manager will resolve. Notable features that VeloxChem offers with the help of other packages include the conversion of SMILES strings to xyz-coordinates with RDKit,^18^ spectrum plotting with Matplotlib,^19^ force field based MD with OpenMM,^20^ management of MD trajectories with MDAnalysis,^21^ and the conversion between Cartesian and internal coordinates as well as the calculation of vibrational frequencies from the Hessian with geomeTRIC.^22^

In Figure 1, we present key classes in VeloxChem that are used to build simulation workflows. We broadly categorize them as associated with the phases of (i) building model systems, (ii) obtaining force field parameters for classical regions, and (iii) calculating the properties of interest. In the subsequent sections, we describe and exemplify these selected classes in some details with the aim that users should be able to create modified and extended workflows following their own needs.

**Figure 1:**
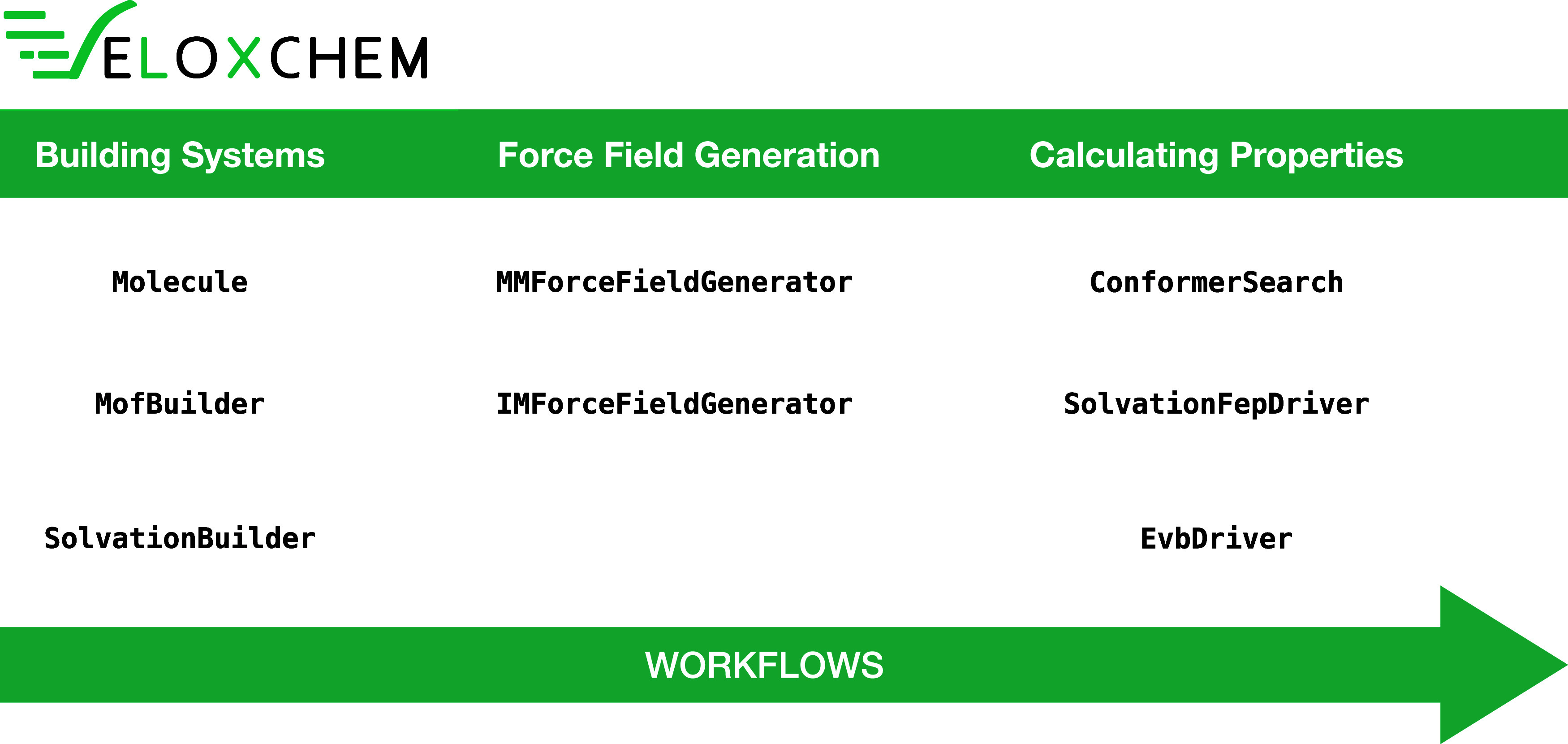
Key classes in the VeloxChem program for the creation of workflows and automatization of simulations.

### Building Systems

#### Molecule

The Molecule class is central to VeloxChem and instances of this class host the QM regions of all model systems. Among the multiple methods to instantiate this class, we note the opportunities to (i) read files in xyz or PDB format and (ii) read strings in xyz or SMILES format.

Once created, a molecule object can be viewed in 2D and 3D representations with the built-in methods draw_2d and show, respectively. The molecular charge, the spin multiplicity, and the structure associated with the object can be modified as to, for instance, alter the initial conformer obtained from a SMILES string with use of the set_dihedral_in_degrees method.

#### SolvationBuilder

The SolvationBuilder class offers effortless solvation of molecules. It creates solvated systems by adding a solvent around a solute molecule defined in the form of a molecule object, and it supports the standard SPC/E and TIP3P models of water as well as a collection of models for commonly used solvents in chemistry including ethanol, methanol, acetone, chloroform, hexane, toluene, dichloromethane, benzene, dimethyl sulfoxide, tetrahydrofuran, and acetonitrile. In addition, there is the option to use the solute itself as the solvent as to create a pure liquid, or to define a second molecule object and specify it as the solvent—a versatility that will allow simulations of a wide range of chemical environments.

The solvation process is done with the solvate method and begins with loading the solute molecule and centering its coordinates within a cubic simulation box. The dimensions of this box are determined based on the size of the solute and a specified padding distance. Thereafter, solvent molecules are added to the simulation box using a random batch insertion algorithm with a *k*-d tree overlap check^23^ as to reach a targeted solvent density based on the accessible volume within the simulation box where the solute volume is excluded from the total volume of the box. If the solute molecule has a net charge, counterions (Na^+^ or Cl^–^) are added to neutralize the system. Optionally, the system can in the end be equilibrated by means of an energy minimization followed by a short MD run in the *NPT* ensemble.

#### MofBuilder

Metal–organic frameworks (MOFs) represent a wide range of crystalline porous materials that combine inorganic secondary building units (SBUs) with organic linkers. This combination gives MOFs an exceptional tunability with several promising applications and tremendous potential, but they are also associated with diverse reticular structures that are not easily accessible without well-structured crystallographic data as the starting point for the building of model systems.

To facilitate the construction of MOFs in a near automatized fashion, allowing for systematic system variations, VeloxChem implements the MofBuilder class. Commonly used SBUs and famous MOF families are supported and user provided organic linkers will be processed as to fit into the framework. The building options of MOF models include customizable topologies, defects, dummy metal atoms in SBUs, flexible terminations for cleaved SBUs, mixed linkers, mixed SBUs, and more.

In the end, a MOF supercell model with selected SBU terminations is created, and further defects can be introduced at this stage. The MofBuilder is readily combined with the MMForceFieldGenerator class described later as to create an MM force field in preparation for MD simulations. The model preparation time for MOFs is thereby dramatically reduced, and in combination with the recently presented state-of-the-art GPU-accelerated implementation of DFT in VeloxChem,^24^ we present a significant advancement of the tool set made available for studies of the molecular and electronic structures of MOFs.

### Force Field Generation

#### MMForceFieldGenerator

The initial step in the automatized creation of an MM force field is to determine the topology of the molecule. The MMForceFieldGenerator class assigns to every atom in the molecule its general AMBER force field (GAFF)^25^ atom type and an initial MM force field is created based on the associated force field parameters for bonded (stretch, bend, and torsion) and nonbonded Lennard-Jones interactions. For elements not covered by GAFF, the universal force field (UFF)^26^ is employed as a fallback. In addition, chemical atom equivalency is detected at this stage, and information that is used for the calculation of the restrained electrostatic potential (RESP) charges that enter into the nonbonded interactions of the force field.^27^

In cases when the MM force field parameters for bond and angle stretching are poor or nonexistent, e.g., in organometallic systems, they can be obtained from the QM optimized structure and the associated molecular Hessian with the use of the Seminario method.^28^

Highly reliable force fields are essential for many applications and in particular torsional motions can be problematic with standard parameters that fail to reproduce true rotational potentials and barriers. In such cases, the automatized MM force field generation in VeloxChem excels, as it employs the reparametrize_dihedrals method to fit the MM force field against QM reference data obtained from relaxed scans. Alternatively, it is possible to supply external reference data in a file if one, for instance, wishes to fit parameters against data obtained with an electronic structure theory method that VeloxChem does not provide.

The internal QM dihedral scans are performed at *N* evenly spaced points in a range of angles, and the fitting is performed against a potential of the form

1 $$V\left(\phi \right)=\sum_{i=1}^{n}A_{i}\left[1+\cos\left(p_{i}\phi -\phi_{i}\right)\right]$$

where *A*_*i*_ are the barrier heights, *p*_*i*_ are integers determining the periodicity, and ϕ_*i*_ are phase shifts. The introduced potential parameters are determined by means of the least-squares fitting of MM energies against the QM reference data, and the resulting energy profiles can be plotted for a visual assessment.

#### IMForceFieldGenerator

The interpolation framework within VeloxChem provides an automated approach to construct customized interpolation mechanics (IM) force fields for molecular dynamics simulations. This framework is based on interpolation methods in which the potential energy surface (PES) is approximated using a weighted sum of the Taylor expansions of the potential energy.^7^ The weighting of an individual potential contribution is determined as a function of the distance between the Taylor expansion center and the position where the potential energy is evaluated, and the Euclidean distance defined in the Cartesian phase space is employed in VeloxChem.^29^ Two types of weighting functions are distinguished: a simple one-part weighting function, which is inversely proportional to an integer power of the distance, and a more sophisticated modified Shepard-like function that consists of two components.^7,30^ Due to the simplicity of the underlying mathematical forms and their strong correlation with individual data points, this method offers an intuitive and practical approach to generating customized force fields for accurate dynamic simulations.

The accuracy of the generated PES depends primarily on the number of reference points where the Taylor expansions are centered, and it generally improves as more points are included. Consequently, this framework enables the automated and efficient generation of highly accurate force fields for a wide range of molecular systems. Within the framework, the fundamental philosophy of primary driver classes has been adapted and embedded into the IMForceFieldGenerator class. The central object of the interpolation method is the database of reference points, for which the energy, gradient, and Hessian must be computed in Cartesian coordinates. The construction of such databases is attained by the compute function, which requires only the molecular structure in the form of a Molecule object as input. Because internal coordinates usually perform better than Cartesians toward increasing the trust radii of Taylor expansions, the compute function initiates the IM force field construction by determining the internal coordinates, energy, gradient, and Hessian for the given molecule, with the derivatives subsequently transformed into the internal coordinate representation which can be determined by the user. Following this, the molecule is integrated into a dynamics simulation environment, where the system is propagated together with the associated potential energy and force calculations based on a set of data points. The database can be expanded with additional structures as the dynamics evolves. Indeed this is essential for improving the IM force field to a desired accuracy and is a key step in the construction of the global PES. Once a preset number of data points is reached or the interpolation accuracy has converged, the construction process terminates, and the final database becomes available as a binary file. Furthermore, it is possible to perform both database construction and dynamical simulations in more complex environments, analogous to the more conventional QM/MM approaches. In this framework, the region normally defined as the QM region is instead described by an IM force field that, in turn, becomes embedded in an MM region, resulting in an IM/MM force field model.^31^ The IM/MM interactions are given by standard nonbonded force field potentials, and the associated parameters are automatically determined with use of VeloxChem in the form of calculated RESP charges and tabulated GAFF Lennard-Jones parameters.

### Calculating Properties

#### ConformerSearch

Since most observable properties relate to the lowest conformations of molecules, it is important to efficiently locate these structures for any computational chemistry problem. The implementation of a robust and automated scheme for conformer search is therefore highly science enabling. The ConformerGenerator class in VeloxChem generates a series of conformations over all possible combinations of dihedral minima. By recognizing rotatable bonds and periodicity, all possible conformers will be generated and optimized by an MM force field. For quite large conformational spaces up to and somewhat beyond some ten rotatable bonds, this method is applicable and will rigorously identify all relevant conformers involving rotations around single bonds outside of cyclic structures.

In addition, when a molecule has charged or polar groups, strong electrostatic interactions may dominate and distort conformer optimization. Implicit-solvent methods can effectively dampen those electrostatic forces by embedding the solute in a continuum, smoothing the energy profile. They treat discrete solvent molecules as a uniform dielectric medium, making them computationally affordable and efficient. The generalized Born (GB) model and its variants^32^ are widely used in MD and can be deployed in conformer generators. They use an effective Born radius and a centered point charge to represent each atom in the molecule, then calculate pairwise electrostatic screening based on the interatomic distances and the corresponding Born radii. The user can use the show_available_implicit_solvent_models method to check all available implicit-solvent models and reset the dielectric constant in the chosen model to switch from the default water to another solvent.

With increasing molecular size, the search for conformers quickly becomes a tedious and time-demanding task. Moreover, the rotatable bonds based approach cannot sample ring conformers in its present version. VeloxChem, therefore, also offers another alternative based on molecular dynamics that is made available in the OpenMMDynamics class. By using the conformational_sampling method, molecular conformations are found by performing an MD simulation at high-temperature to overcome dihedral barriers in the force field, and taking snapshots from this trajectory that are in turn MM structure optimized. The user can define the number of snapshots to be taken as well as the time length of the MD simulation. The Boltzmann distribution of the generated conformers can subsequently be determined using the calculate_boltzmann_distribution method.

Regarding both approaches, unique conformer structures are identified based on a comparison of energies and structure similarities. Optionally, the set of conformers found to be lowest in energy can be QM structure optimized.

#### SolvationFepDriver

The free energy of solvation, quantifying the thermodynamic change associated with transferring a molecule from vacuum to a condensed phase, plays a pivotal role in our understanding of biological activity, drug solubility, and various phenomena in materials science. The SolvationFepDriver class in VeloxChem provides an efficient and automated workflow for calculating free energy of solvation using explicit solvent molecules in molecular dynamics simulations. The class implements a free energy perturbation (FEP) method, simulating a series of nonphysical intermediate states. This approach provides an alchemical pathway through which the solute can be transferred from vacuum to solution, and it ensures a sufficient overlap in phase space between the neighboring states that is crucial for accurate free energy evaluation. The states are constructed by linearly scaling the solute–solvent interactions using a coupling parameter λ. The total free energy difference is then calculated by summing the energy differences between each intermediate state. Addressing end point singularities in FEP calculations, which cause convergence issues from energy fluctuations during solute appearance/disappearance, the SolvationFepDriver class employs an *ad hoc* Gaussian potential.^33^ Unlike conventional separation-shifted soft-core potentials, this approach mitigates end point singularities while preserving a linear λ-dependence. This has demonstrated to improve computational efficiency, enabling accurate free energy estimates with fewer λ-states.

The class workflow encompasses (i) solvating the molecule with the SolvationBuilder, (ii) generate separate force fields for each λ-state, (iii) equilibrating and running simulations for each state, and finally, (iv) calculating the energy difference between each state using the multistate Bennett acceptance ratio (MBAR) method.^34^ This fully automated workflow requires minimal user input, typically only the molecule and solvent (if other than water) for the compute_solvation function. Other solvents can be introduced either by choosing among the available selection in the SolvationBuilder class, or by defining solvent Molecule object(s). For increased flexibility, users can provide their own solute and solvent force fields in either OpenMM or GROMACS format using compute_solvation_from_omm_files or compute_solvation_from_gromacs_files, respectively. The protocol can be customized for more complex systems e.g., by adjusting simulation length, introduce more lambda states or include more samples from each simulation in the final MBAR calculation.

#### EvbDriver

The empirical valence bond (EVB) method^35^ is a powerful method to determine the free energy profile of a reaction pathway in different electrostatic environments, and it has been successfully used to describe the reactivity in complex systems such as enzymes^36^ and electrocatalysts.^14^ The method revolves around performing free energy perturbation calculations of the reaction in different environments by linearly interpolating from a reactant to a product force field. The energy profile of one environment is then reparameterized using reference values for the reaction barrier and reaction energy to account for the energy change due to the breaking and forming of chemical bonds. This parametrization of the free energy profile generalizes to other environments from which accurate reaction barriers and energies can be obtained. The difficulty of performing EVB calculations lies mainly in accurate interpolation between the reactant and the product as this is practically difficult in most existing software. Recently, an EVB workflow was demonstrated using GROMACS,^37^ where the documented workflow consisted of 18 separate manual steps. This is largely due to the fact that GROMACS, like most other MD software, is not built for the simulation of breaking and forming bonds.

VeloxChem presents a completely automated and customizable workflow. This functionality is organized in the EvbDriver class. The default workflow uses a vacuum configuration as reference to obtain a free energy profile for the reaction in water. The user supplies structures for the reactant and the product, and the reaction barrier and free energy in vacuum. From there, the program takes care of everything else. If more control is desired, the user can provide a force field, specify different solvents, electric fields, and adjust the parameters for the FEP calculation. Another feature of our implementation is the possibility to perform an FEP calculation in the same environment at different temperatures from which the activation enthalpy and entropy can be computed as demonstrated in ref 37.

## Illustrative Examples

The design of the aforementioned classes has been centered around user experience, with the aim of lowering the barrier for experimentalists and quantum chemists to employ molecular mechanics tools valuable for exploring chemical reactivity. To illustrate their practical application, we present several realistic examples of calculations along with code snippets containing the key code lines taken from the self-contained Jupyter notebooks provided in the Supporting Information.

### Building Systems

#### Molecule Object

Molecule objects can be created in a number of ways out of which we here demonstrate three alternative methods namely reading (i) SMILES strings, (ii) xyz-files, and (iii) xyz-strings. The SMILES-string approach includes a structure optimization employing the universal force field (UFF) but does not perform any conformer search as to find the conformer lowest in energy.

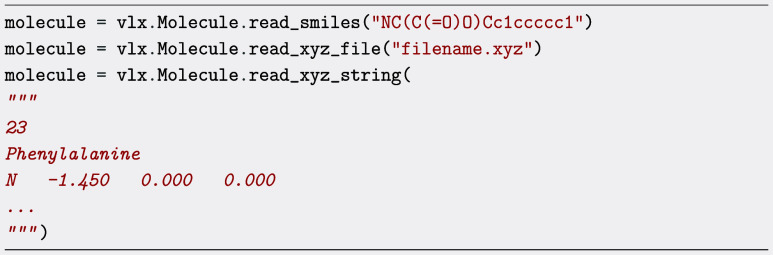



Basic modification of the molecule object can be performed, such as setting the total charge and modifying values of dihedral angles. The latter functionality offers a convenient way to alter conformers. It is recommended to always do a visual inspection of the molecule before starting further calculations.

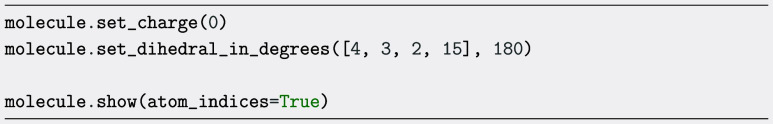



#### Metal–Organic Frameworks

VeloxChem features a builder for studies of MOFs whose versatility is illustrated here using a selected linker (dcphOH-NDI^38^) to build a UiO-66 analogue. The metal type in the SBU node is selected as Zr. After the standard model building workflow, a defective model with missed linkers is created by listing the indices of linkers to be removed. The index of linkers, SBU nodes, and other units in the MOF model can be found from the output file of the standard MOF model. The visualization of the built UiO-66 analogue and its defective model are shown in Figure 2.

**Figure 2:**
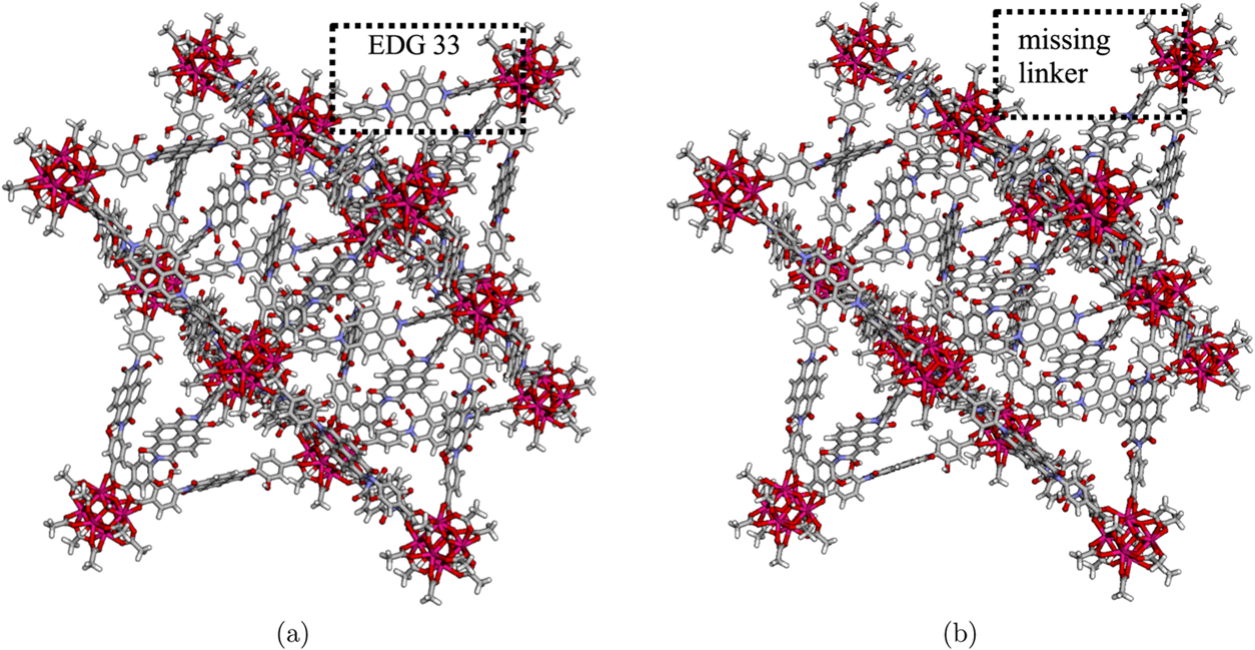
Visualizations of the built (a) UiO-66 analogue model with dcphOH-NDI as linker methyl group as cleaved Zr SBU node termination and (b) defective UiO-66 analogue with a linker missing. Visualization has been obtained with VIAMD.^39^

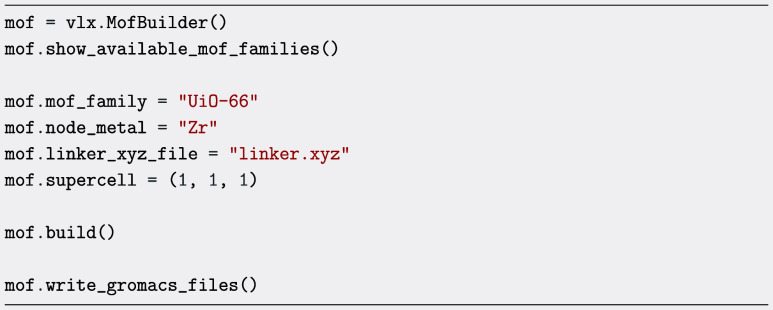



Defects can be introduced by removing linkers or nodes. For example, we can remove a set of linkers with indices collected in a list as illustrated in Figure 2b.

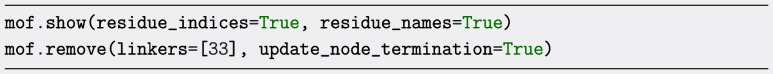



#### Solvation

Below is an example of how to use the SolvationBuilder class to solvate a molecule.

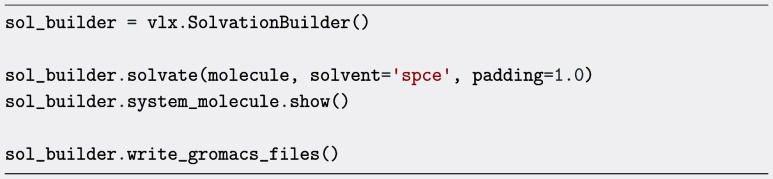



Only default settings are applied in this code, resulting in the creation of a box with a 1.0 nm padding filled with a noradrenaline molecule and water molecules, as demonstrated in Figure 3. GROMACS-compatible files necessary for running dynamics are written for the solvated system using write_gromacs_files. An analogous write_openmm_files function is available as well. To accommodate more complex systems, such as mixed solvents, the custom_solvate function can be employed. This function allows users to define a list of solvent molecules and their respective quantities, offering enhanced flexibility in the system preparation.

**Figure 3:**
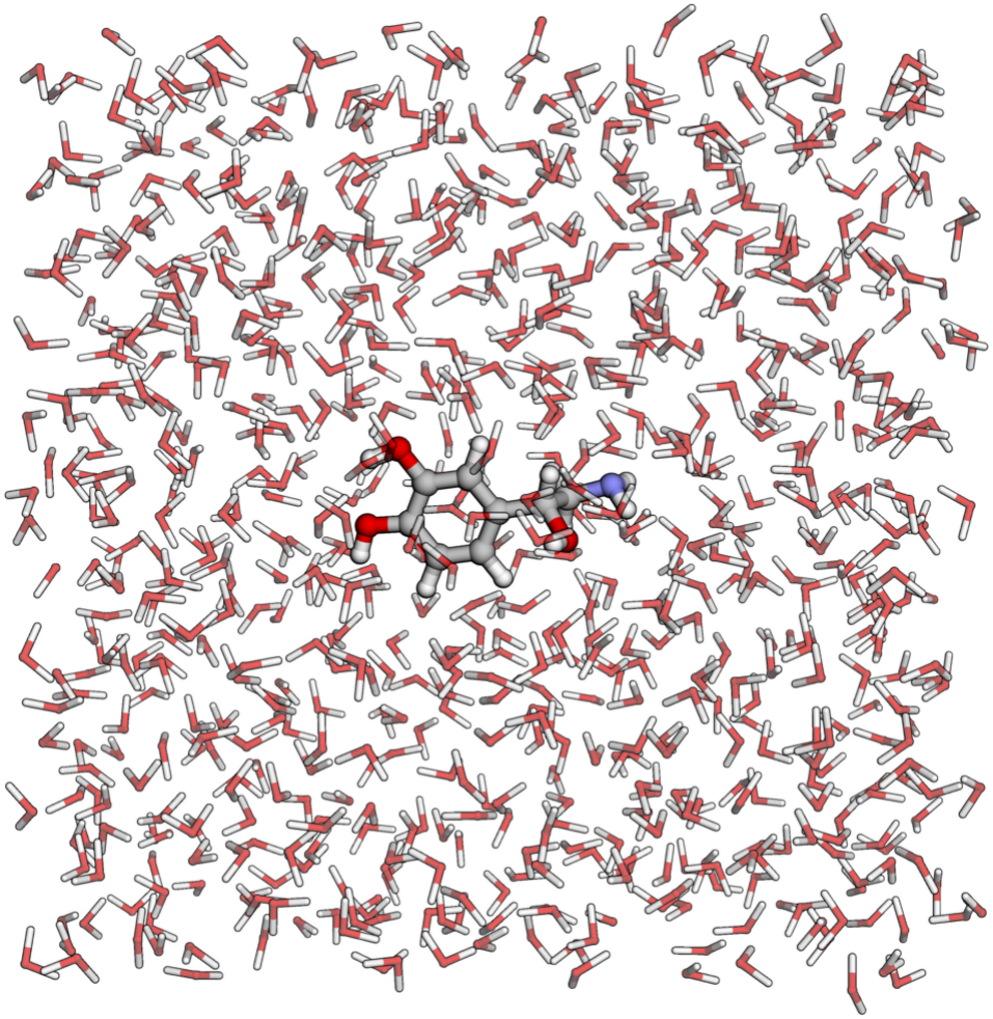
Illustration of noradrenaline solvated in a box of water with a 1.0 nm padding of the solute.

### Force Field Generation

#### Molecular Mechanics

The creation of MM force fields is fully automatized in VeloxChem and made available through the create_topology method. RESP atomic charges are calculated at the HF/6–31G* level of theory by default. Alternatively, the topology can be provided with either semiempirical charges, or a list of user-defined atomic charges.

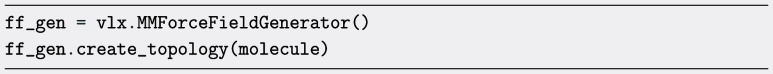



The resulting force field can be exported for use together with the GROMACS and OpenMM programs.

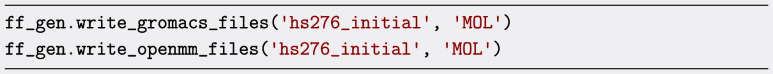



The force field quality can be readily assessed by visual inspection. The most critical part is associated with the tabulated GAFF force constants for dihedral rotations, and it is recommended to always check the quality of the rotational barriers. A listing of dihedrals is available with use of the rotatable_bonds method.

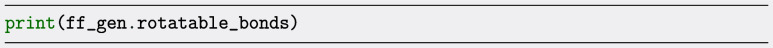



The key rotatable bond for this ligand is identified in its intended application as a fluorescent probe for the detection of amyloid protein aggregates.^5,40^ We get a relaxed scan potential energy curve (PEC) for this bond at our specified QM level, either in the Jupyter notebook or with use of a cluster.

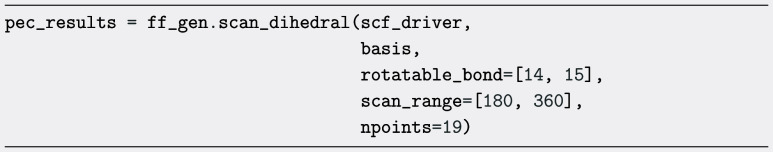



The comparison of the QM and MM potential energy curves is readily made with the implemented plotting methods as shown in Figure 4a for HS-276,^41^ a ligand that selectively targets amyloid-β deposits as one of the pathological hallmarks in Alzheimer’s disease. It is noted that the rotational barrier is clearly too high and the conformer minima are not well reproduced by the MM force field.

**Figure 4:**
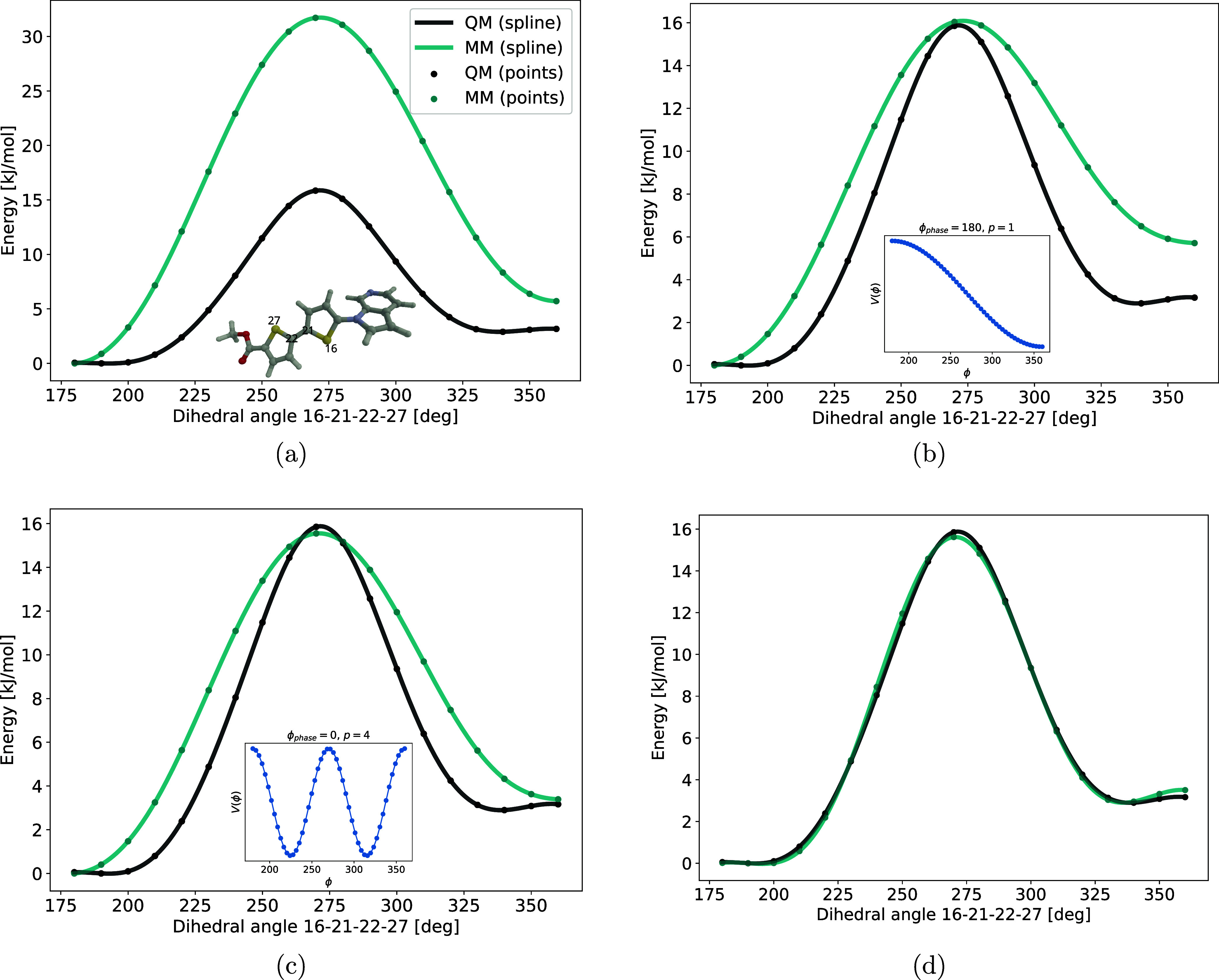
Force field parametrization of HS-276: (a) initial force field prediction in green and QM reference data in black. (b) Results after fitting MM energies to QM reference data and showing a first dihedral in the inset. (c) Results after adding the first dihedral and showing a second dihedral in the inset. (d) Results after adding the second dihedral.

To improve the MM force field in this respect, we make use of the reparameterize_dihedral method that performs the reparameterization of the dihedral force constants associated with this given rotation.

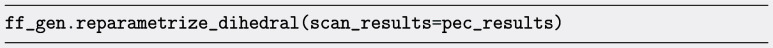



The reference data for the reparameterization is provided in this example by the results dictionary, but it is also possible for the user to instead provide a scan file with reference data with an argument of the form scan_file = ″16–21–22–27.xyz″. The user can specify fit_extrema = True to exclusively consider the extrema (maximum and minimum points) in the parametrization; however, in most cases, including all data points yields a better fit. The resulting reparameterized MM force field is shown in Figure 4b. The rotational barrier is now much improved but the relative energies between the two conformer minima are still not well reproduced, leading to significant errors in the statistical dihedral distributions.

As a remedy to this situation, an additional dihedral potential shown in the inset of Figure 4b is added to the force field.

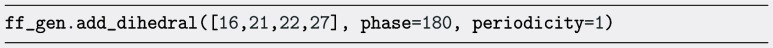



Here, the function arguments phase and periodicity correspond, respectively, to the ϕ_*i*_ and *p*_*i*_ parameters introduced in eq 1. After a refitting is performed, the resulting MM potential is shown in Figure 4c, demonstrating that the MM potential is still not in perfect agreement with the QM reference potential. Yet another dihedral potential shown in the inset of Figure 4c is therefore added.

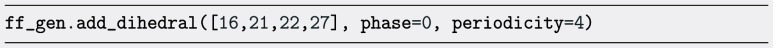



After a refitting is performed, the resulting MM force field is now seen to be in excellent agreement with the QM reference potential, see Figure 4d.

The user can sequentially repeat this process for all key rotatable bonds for the functioning of the ligand. As force field parameters are generally interdependent, it is advisable to repeat the procedure and go over all the dihedrals in a second iteration—it is, however, our experience that convergence is quickly reached—as to end up with a highly accurate MM force field at the end of the workflow. In this way, we have found it possible to sample ligand configuration spaces sufficiently accurately for applications in spectroscopy in the condensed phase.^5,42,43^

#### Interpolation Mechanics

This section demonstrates the user-friendly functionality of the IMForceFieldGenerator class. The construction of an IM force field merely requires the specification of a QM method and the standard molecule and basis objects. Once these are defined, the force field database can be constructed with the compute method without further specifications, should the user settle for the default settings. The return dictionary from this method contains information about the number of data points in the database, which by default is given the name im_database.h5 but can be user specified.

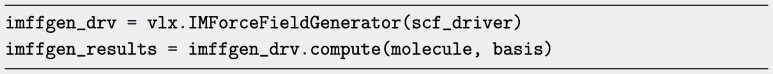



The confirm_database_quality method allows the user to confirm the quality of the IM force field and, if needed and requested, expand the database to improve the quality. The quality assessment function is applied at the end of each database construction process, but can also be utilized for an externally defined database, requiring only a molecule object and the corresponding database file as input. The database is then examined against a predefined energy threshold. If the interpolation energy of individual structures from a test simulation deviates above a threshold value, then the database is expanded to include those structures. This process is iteratively repeated until convergence is reached.

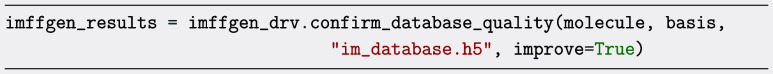



The add_point function is used to generate a new data point for a given molecular structure and add this data point to the database.

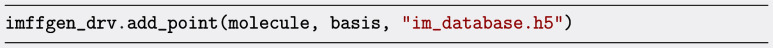



As an example in Figure 5, we demonstrate the creation of an IM force field for bithiophene. To begin with, we created a molecule object and identified the dihedral angle (3–4–6–10) that is the key internal coordinate for the creation of this force field. The database construction was carried out in the form of a scan as follows: First, ten relaxed structures were generated equidistantly over the full 360 deg rotation of the dihedral angle, and these structures were added one at a time to the database. With each added point to the database, a dynamics simulation was performed with dihedral displacements restricted to ± 18 degrees—this approach ensures that the immediate environment of each structure was well sampled and adequately described. During these dynamics simulations, an energy threshold of 8.4 kJ/mol was applied and whenever a sampled structure showed an IM–QM energy difference above this threshold, the structure data was added to the database. A given dynamics simulation was terminated if no new data points were added during a 7.5 ps time of simulation. The final database constructed this way comprised 12 data points. As shown in Figure 5, this iterative procedure resulted in an accurate and well-sampled representation of the key dihedral angle in bithiophene, demonstrating the feasibility of automatically constructing reliable force fields with minimal user intervention. This approach holds particular promise for the development and application of multistate force fields, enabling the simulation of accurate multistate dynamics, which is currently under development and will be available in the near future.

**Figure 5:**
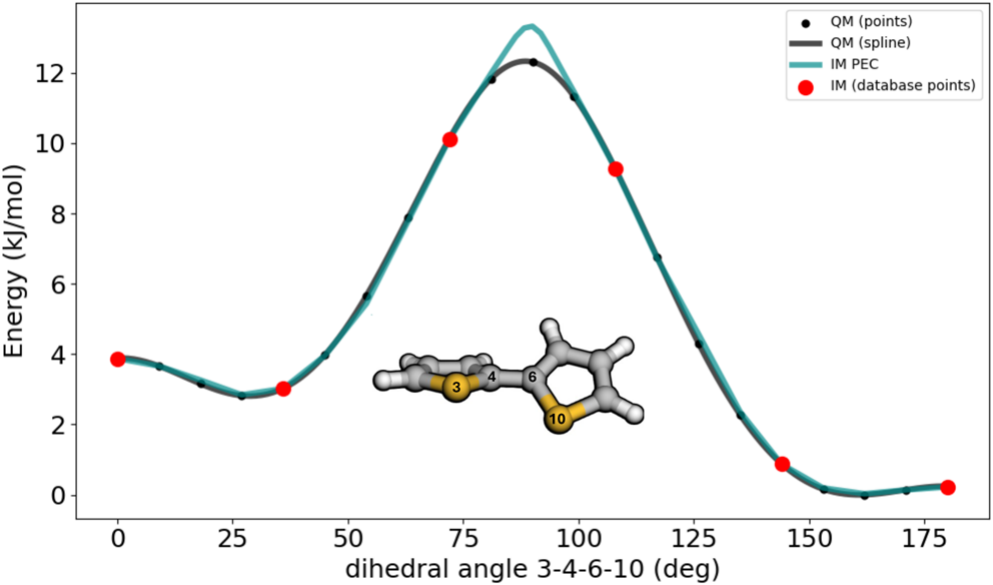
Comparison of QM and IM potential energy curves for the S–C–C–S dihedral rotation in bithiophene. The PEC coordinate is that associated with the QM relaxed scan at the B3LYP/def2-svp level of theory.

### Calculating Properties

#### Conformer Search

To generate all unique conformers with respect to rotatable bonds, we execute the generate method in the ConformerGenerator class.
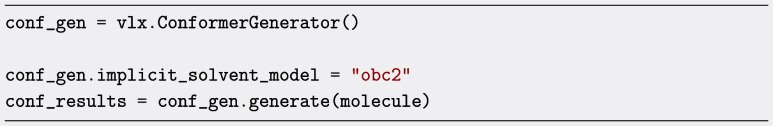


The implicit OBC2 solvent model^44^ of water is here used for the MM energy minimization of all generated configurations—some 2,304 in total for osimertinib as a result of eight 2-fold rotatable bonds and two 3-fold rotatable bonds (leaving out methyl groups). This system is also known under the drug name tagrisso. After structure optimization of the generated configurations, some 719 unique conformers remained. The results for the unique conformers are returned as a dictionary containing MM energies and molecule objects that can be used directly for other VeloxChem operations. In this case, the 97 most stable conformers were selected for more accurate QM optimizations as to cover relative MM energies up to some 20 kJ/mol, see Figure 6.

**Figure 6:**
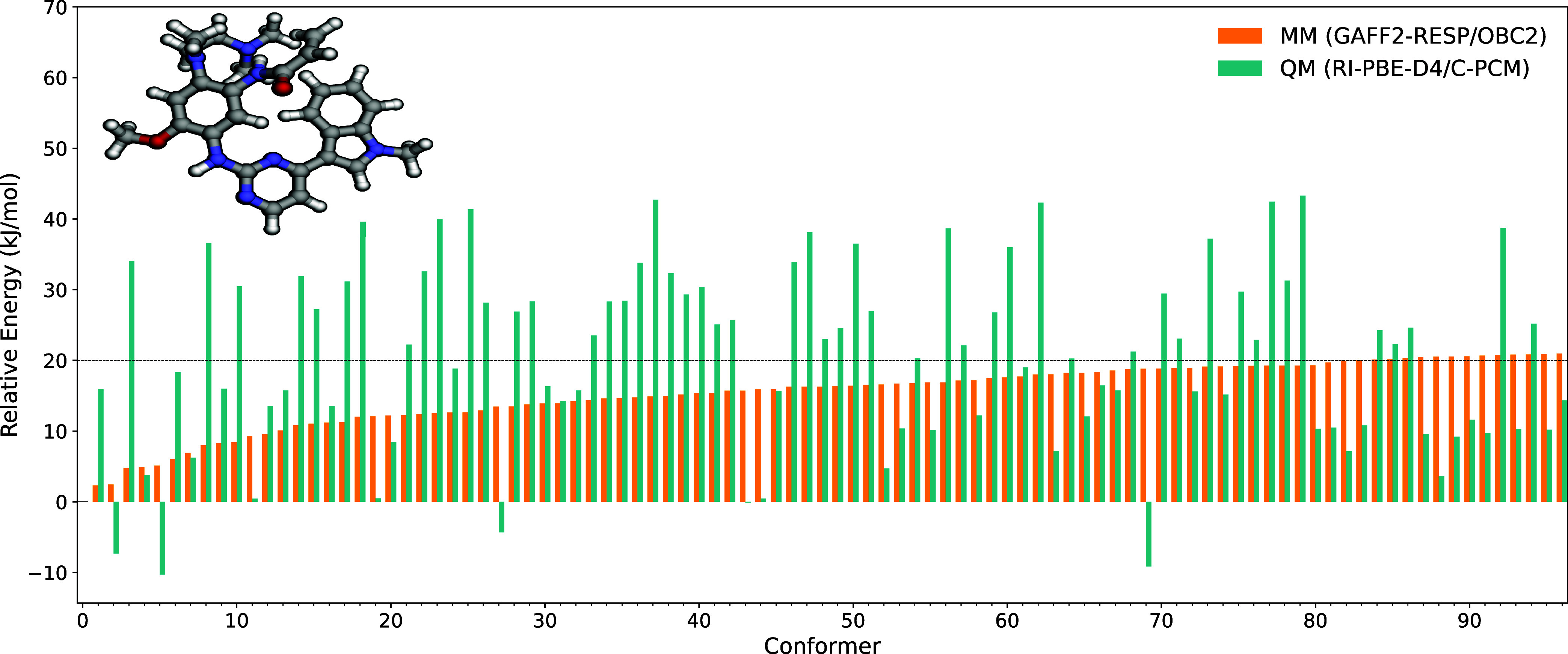
MM and electronic QM energies of the lowest optimized conformers of osimertinib in water using implicit solvation models. The conformers are ranked by the MM results.

At the QM level, the most stable conformer was found to be number five, closely followed by number 69, which demonstrates the fact that the MM energies are not sufficiently reliable for accurate predictions of relative conformer energies but merely serve the purpose of screening. The employed force field is obtained with the MMForceFieldGenerator class described above, i.e., essentially tabulated GAFF parameters in combination with calculated RESP atomic charges. As illustrated in Figure 4, such a force field can be of limited quality when it comes to the description of dihedral rotations and a reparameterization of this part of the force field typically needs to be carried out.

To generate conformers based on high-temperature MD simulations, the method

conformational_sampling from the OpenMMDynamics class is employed.
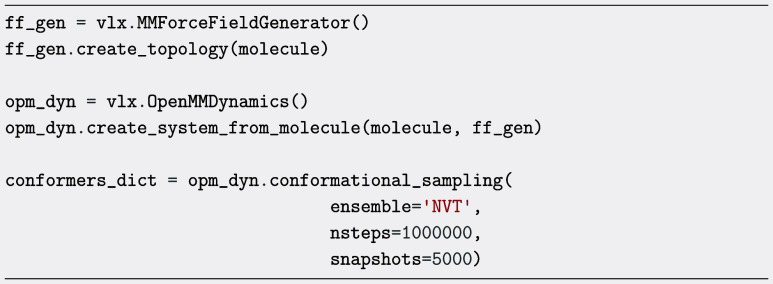


As an illustrative example, we have chosen the case of a segment of a thiophene quinoxaline polymer that contains thirty-one 2-fold rotatable bonds and 16 3-fold rotatable bonds (leaving out methyl groups). Herein, 5,000 snapshots are collected and structure optimized from a 2 ns simulation performed at the default temperature of 700 K. Postsimulation, identical conformers are filtered out, retaining only unique structures and which in this case resulted in 4,815 conformers. The function then returns a dictionary containing the conformer energies, molecular objects, and corresponding Cartesian coordinates, all sorted in ascending order of energy. The wide distribution of conformer energies obtained by this approach is depicted in Figure 7. Four conformers within 5 kJ/mol have been identified, the most stable conformer being represented as an inset of the figure.

**Figure 7:**
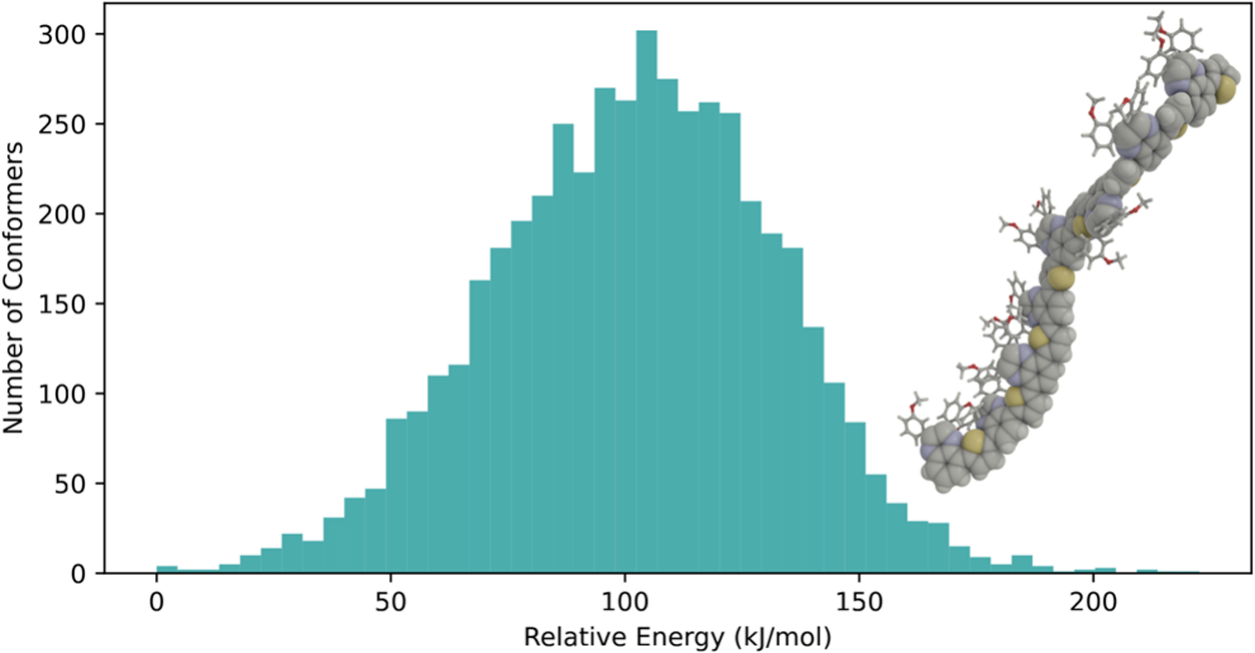
Conformer energy distribution for a thiophene quinoxaline oligomer based on 4,815 unique conformers obtained from a high-temperature MD simulation. The structure of the most stable conformer is shown in the inset.

#### Free Energy of Solvation

The following code snippet serves to illustrate the highly automatized protocol for performing free energy of solvation calculations, provided in the SolvationFepDriver in VeloxChem. Using all default settings, the user only needs to define the Molecule object, instantiate the class, and call the compute_solvation function to begin the calculations with water as solvent. The function will return a dictionary with information about the energy and statistical uncertainty from each step in the protocol, along with the final free energy of solvation.
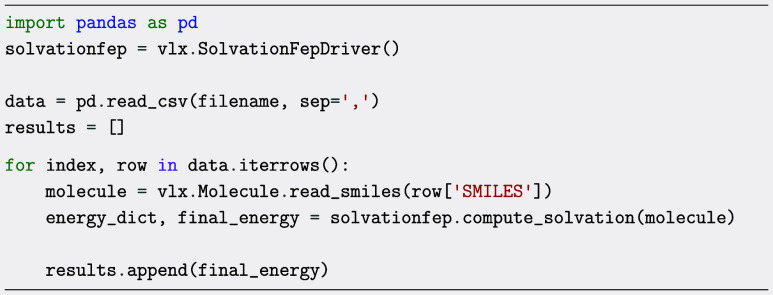


In the above example, the solvation free energies in water are computed for a set of 53 small molecules obtained from the database reported in ref 45. The data set comprises both neutral and charged species, spanning a range of different chemical classes. Calculations are carried out by iterating over the SMILES strings for each molecule. To account for the Galvani surface potential, associated with transferring an ion across the vacuum–bulk solvent interface, a correction term of –*qϕ*_G_ is applied to all computed solvation free energies of ionic species, following the approach described in ref 46., where *q* denotes the formal charge of the molecule. The parameter ϕ_G_ is solvent model-dependent and, for the SPC/E water used in these simulations, corresponds to −57.7 kJ/mol per unit charge. Computed solvation free energies are compared to experimental reference data in Figure 8.

**Figure 8:**
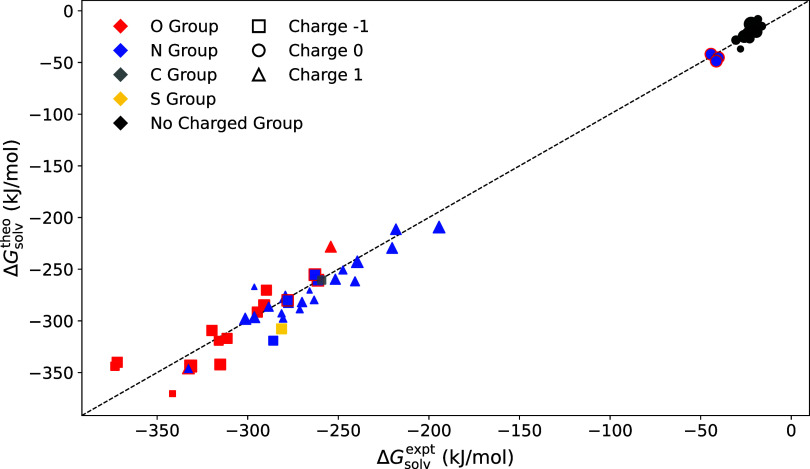
Comparison of theoretical and experimental results for solvation free energies for a data set taken from ref 45. Galvani’s potential corrections are applied to all ionic species. Marker size reflects the relative radius of gyration (0.92 ≤ *R*_gyr_ ≤ 2.86 Å). Marker shape denotes the net molecular charge. Marker color indicates the functional group(s) carrying the charge. Zwitterions are represented by markers with multiple colors.

Evident from Figure 8, neutral systems exhibit less negative solvation free energies compared to charged species. Among the ionic compounds, anions tend to display more favorable solvation than cations. This trend is further reflected within the functional group-specific behavior. The oxygen-containing groups are negatively charged in all cases except one: the OH^+^ group in 1-phenylethylideneoxidanium, which carries a positive charge and exhibits the least favorable solvation among the oxygen species. Similarly, amine-based groups are generally positively charged, with the exception of the NH^–^ group in phenylazanide, which is negatively charged and displays a more favorable solvation compared to the other species in the same group. These exceptions reinforce the broader trend that anionic species are more strongly solvated than the cationic counterparts. The data set also includes a number of zwitterionic compounds, recognized by the presence of multiple functional group color in the plot. Zwitterions with a net formal charge of zero are classified as neutral, while those with unequal number of positive and negative ionic groups are classified as charged species.

#### Empirical Valence Bond Potentials

The following code snippet demonstrates how to run the EVB protocol for an S_N_2 reaction in a series of environments. As an illustration, we chose the well-known reaction between chloromethane and a bromide anion in vacuum, water, dimethylformamide, and acetone. The input consists of two xyz-files, although molecule objects can also be used. The code automatically constructs the force fields and performs any necessary reparameterization. It then constructs the systems describing the different environments after which it performs the FEP calculations for all different environments. Lastly, it fits the energy profile of the first environment (vacuum in this case) with user supplied parameters that in our case were obtained from ref 47, and calculates the free energy profiles for the other cases.
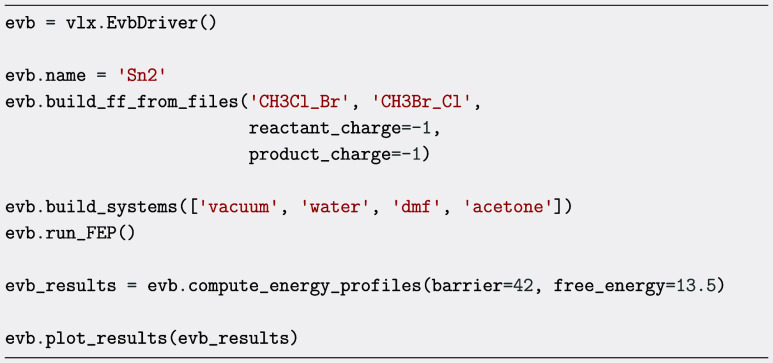


With default options, the FEP calculations are done with 61 frames over a total of 6.1 ns excluding equilibration. If necessary, it is possible to adjust the pressure, temperature, different solvents, periodic box size, and FEP parameters such as equilibration times and the λ-vector. The results of the calculation are returned in a dictionary that can be plotted with a built-in method as shown in the code snippet, see Figure 9. As expected, any solvent stabilizes the reactant and product states, leading to a higher reaction barrier for all solvents compared to vacuum. Furthermore, the product state with the nonbonded chloride ion is stabilized significantly more in water than in the other, aprotic, solvents. This stabilization of the product in water is a consequence of the strong hydrogen-bonding interaction between water and the chloride ion, whereas the bromide ion forms weaker interactions with water.^48^

**Figure 9:**
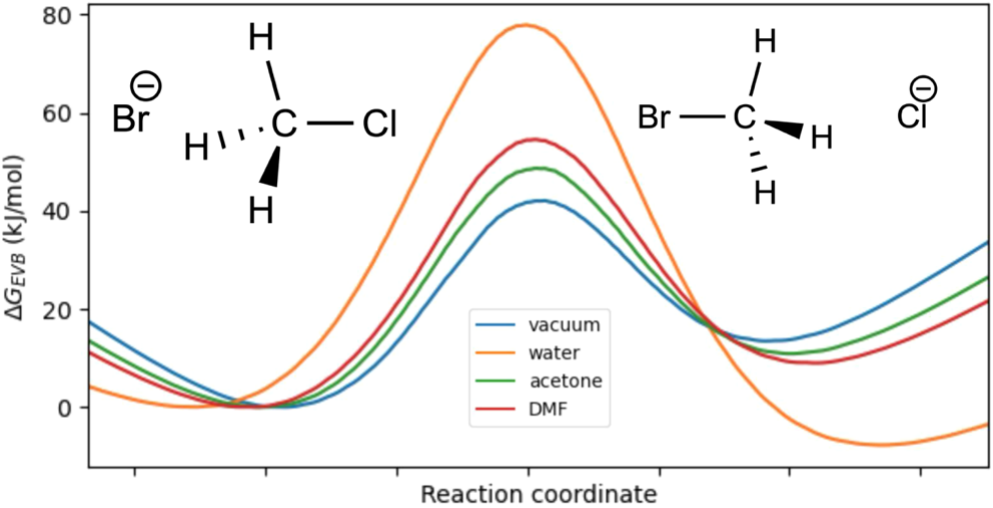
Free energy profiles of bromine substitution of chloromethane in various solvents. All profiles are shifted so that the reactant energy is set to zero, and the vacuum barrier and product free energy are parametrized to reference values.

## Concluding Remarks

By now, it is well recognized that there are a number of benefits that come with the adoption of the Python programming language for the development of most parts of quantum chemistry software.^17,49–51^ With a good software design, it leads to a quantum chemistry simulation environment that is intuitive and easy to use, valuable in education,^52,53^ flexible and easy to adapt after specific needs, associated with a low barrier for development,^54^ and easy to combine with external modules such as e.g., PyFraME^55^ to facilitate QM/MM modeling of complex systems.

There is still, however, a noticeable gap to be found between software developed in the quantum and classical communities of theoretical chemistry. It is made clear that the quantum community also adopts classical methods e.g., in various embedding models, but with the classical community we here refer to developers of methods and software for force field driven molecular dynamics. Simulations of complex systems in real applications of materials science and biochemistry typically require the use of software from both these communities. At the same time, users are left on their own to figure out ways how to best achieve a combined usage of programs and solve the issue of data transfer in between them.

The present work argues that we can reach a leap in simulation technology and scientific progress by filling this gap and strive for true software interoperability with data transfer using internal data types rather than files. We demonstrate how such an approach provides the opportunity to construct automatized workflows of simulations in chemistry that otherwise require significant expertise and experience. It can be said that there is a risk of providing black-box access for nonexperts to rather complicated methodologies, but we would argue that this is a sacrifice we must be willing to pay for the advancement of technology, be it concerned with chemistry simulations or something else.

## Supplementary Material



## Data Availability

The presented code snippets are taken from the Jupyter notebooks. These notebooks, together with data files upon which they depend, are also available in electronic form for download from the workflow folder of the following GitHub repository: https://github.com/VeloxChem/vlx-notebook.git.

## Notes

Supporting Information: Jupyter notebooks demonstrating how to (i) build a molecule object, (ii) build a metal–organic framework and generate a force field, (iii) generate a solute force field and solvate the system, (iv) determine free energies of solvation, (v) fit MM force field parameters, (vi) generate an IM force field, (vii) find and rank conformers, and (viii) construct EVB potentials (PDF)
