# Supplementary material for: VeloxChem Quantum–Classical Interoperability for Modeling of Complex Molecular Systems
Source: J Phys Chem A. 2025 Aug 4;129(32):7575–87. doi: 10.1021/acs.jpca.5c03187 (PMC12359120; doi:10.1021/acs.jpca.5c03187)
Supplement: Supplementary file 1 [file jp5c03187_si_001.pdf]

Supporting Information: VeloxChem Quantum-Classical Interoperability for Modeling of Complex Molecular Systems

Juan Angel de Gracia Triviño,[†] Iulia Emilia Brumboiu,[‡] David Carrasco-Busturia,[¶] Xin Li,[†] Chenxi Li,[¶] Mathieu Linares,[†] Valentin Lindfeld,[¶] Young Min Rhee,[§] Julia Rune,[¶] Bastiaan van Hoorn,[¶] Patrick Norman,[*,¶] and Mårten S. G. Ahlquist[¶]

†*PDC Center for High Performance Computing, School of Electrical Engineering and Computer Science, KTH Royal Institute of Technology, SE-100 44 Stockholm, Sweden*

‡*Faculty of Physics, Astronomy and Informatics, Nicolaus Copernicus University in Toruń , 87-100 Toruń, Poland*

¶*Division of Theoretical Chemistry and Biology, School of Engineering Sciences in Chemistry, Biotechnology and Health, KTH Royal Institute of Technology, SE-100 44 Stockholm, Sweden*

§*Department of Chemistry, Korea Advanced Institute of Science and Technology (KAIST), Daejeon 34141, Korea*

E-mail: panor@kth.se

Contents

Section	Page
Building a molecule object	S3
Metal-organic framework builder	S5
Solvation builder	S6
Free energy of solvation	S6
Molecular mechanics force field parameterization	S9
Interpolation mechanics force field parameterization	S11
Conformer Generator	S14
– Systematic generation of all conformers	S14
– Conformer generation with molecular dynamics	S15
Empirical valence bond based free energy potential	S18

Build

Building a molecule object

There is several ways to build a molecule object within VeloxChem using for instance a smile string, directly from an xyz or pdb file or by providing and xyz string. This is explicited below with few examples. The 3D structure of the molecular object can be visualized with the show function, and the atom indices can be shown optionally.

In [ ]:
```
import veloxchem as vlx
```

In [ ]:
```
molecule = vlx.Molecule.read_smiles("NC(C(=O)O)Cc1ccccc1")
molecule.show()
```

In [ ]:
```
molecule = vlx.Molecule.read_xyz_file("data/phenylalanine.xyz")
molecule.show(atom_indices=True)
protein = vlx.Molecule.read_pdb_file("data/15-alanine.pdb")
protein.show()
```

In [ ]:
```
xyz = """
23

N     0.67817   -1.99779    0.02940
C     1.12060   -0.73032   -0.50847
C     0.65228    0.55247    0.25928
C    -0.85069    0.67980    0.32331
C    -1.56734    1.26777   -0.73318
C    -2.96138    1.34750   -0.69980
C    -3.66887    0.83713    0.39385
C    -2.97070    0.25061    1.45250
C    -1.57445    0.17372    1.41602
C     2.64152   -0.72318   -0.52913
O     3.13479    0.08058   -1.49059
O     3.34206   -1.31467    0.25410
H    -0.31939   -1.98039    0.23353
H     1.18324   -2.20594    0.89143
H     0.77321   -0.63212   -1.55101
H     1.08397    1.43374   -0.24202
H     1.07468    0.51182    1.27709
H    -1.02293    1.67365   -1.59118
H    -3.49861    1.81381   -1.52986
H    -4.75954    0.90143    0.42211
H    -3.51376   -0.14475    2.31497
H    -1.03557   -0.27525    2.25529
H     4.10329    0.07185   -1.39601
"""

molecule = vlx.Molecule.read_xyz_string(xyz)
molecule.show(atom_indices=True)
```

Basic modification of the molecule object can be performed such as setting the total charge, mulptiplicity or modifying values of dihedral angles. The latter functionality

offers a convenient way to alter conformers. It is recommended to always do a visual inspection of the molecule before starting further calculations.

In [ ]:
```
molecule.set_charge(0)
molecule.set_multiplicity(1)
molecule.set_dihedral_in_degrees([4, 3, 2, 10], 60)
molecule.show(atom_indices=True)
```

MOF builder

This is a notebook for builidng a Metal Organic Framework (MOF). First, we initialize the `MofBuilder` and show all available MOFs families:

In [ ]:
```
import veloxchem as vlx

mof = vlx.MofBuilder()
# The next line can be skipped if you know the family name
mof.show_available_mof_families()
```

To build a target MOF, one needs to:

1. assign MOF family
2. assign the node metal type
3. upload the linker file (with carboxylate) in xyz format

One can also set the supercell size of the model, then we can ***build*** the MOF, and save the model with gromacs file format, as well as show it:

In [ ]:
```
mof.mof_family = 'UiO-66'
mof.node_metal = 'Zr'
mof.linker_xyz_file = 'data/linker.xyz'
mof.supercell = (1, 1, 1)  # (1,1,1) means primitive cell
mof.build()
mof.show(residue_indices=True, residue_names=False)
```

We can make defective models by removing linkers or nodes. In this example we just want to remove the linker in the corner:

In [ ]:
```
# The index of the linkers or nodes to be removed can be shown
# in the last cell output.
# Remove the linker with index "17, 18, 19"
mof.remove(linkers=[17, 18, 39], update_node_termination=True)
mof.show(residue_indices=True)
mof.write_gromacs_files()
```

SolvationBuilder

In [ ]:
```
import veloxchem as vlx
```

Create a molecule object for deprotonated ibuprofen and generate a forcefield with semiempirical partial charges to use in the following solvationfep calculations.

In [ ]:
```

```

In [ ]:
```
ibuprof = vlx.Molecule.read_smiles('CC(C)CC1=CC=C(C=C1)C(C)C(=O)[O-]')
ibuprof.show()
```

In [ ]:
```
ff_gen_solute = vlx.MMForceFieldGenerator()
ff_gen_solute.partial_charges = ibuprof.get_partial_charges(
    ibuprof.get_charge())
ff_gen_solute.create_topology(ibuprof)
ff_gen_solute.write_gromacs_files('ibuprof','MOL')
```

Load the SolvationBuilder and create a box of padding 1.0 nm (default). Solvate the molecule with water (SPC/E water model). After solvating the box, the SolvationBuilder will run a NPT equilibration of 5 ps at 300K. Once the equilibration is finished, gromacs files for the equilibrated system is written.

In [ ]:
```
solvator = vlx.SolvationBuilder()

solvator.solvate(ibuprof, equilibrate=True, neutralize=False)
solvator.system_molecule.show()

solvator.write_gromacs_files(solute_ff=ff_gen_solute)
```

Free Energy of Solvation

The free energy of solvation for ibuprofen in a mix of water and propylene glycol is computed with the SolvationFepDriver using the gromacs files generated from the SolvationBuilder as input. The functon requires a gro and topology file for both the solvated system and the molecule in vacuum.

In [ ]:
```
solvationfep = vlx.SolvationFepDriver()

# Optional, saving trajectory from each lambda simulation in xtc format
#solvationfep.save_trajectory_xtc = True

# The number of steps here has been chosen for a quick execution in the
# Notebook, we recommend using 500 000 steps for production runs (1 ns)
# per Lambda.
solvationfep.num_steps = 10000
delta_f, final_energy =solvationfep.compute_solvation_from_gromacs_files(
    'system.gro','system.top','ibuprof.gro','ibuprof.top')
```

Custom solvate

Following is an example of how to solvate ibuprofen in a mixed solvent of equal amounts of water and propylene glycol. A VeloxChem molecule object is created for solvent molecules. A list of the solvent molecules, the quantity of each molecule and the box dimension (Å) is specified in the function. Gromacs files for the system is written.

In [ ]:
```
solvator = vlx.SolvationBuilder()

propylene_glycol = vlx.Molecule.read_smiles('CC(CO)O')
water = vlx.Molecule.read_smiles('O')

solvator.custom_solvate(ibuprof,
                        solvents=[water, propylene_glycol],
                        quantities=[200,200],
                        box=(40,40,40))
solvator.system_molecule.show()
solvator.write_gromacs_files(solute_ff=ff_gen_solute)
```

Free Energy of Solvation for a dataset

In this example the free energy of solvation is calculated for a dataset of molecules. The necessary information about each molecule, in this case the SMILES code and charge, is loaded from the *solvationfep_molecules.csv* file.

Iterate over the molecules to run the solvationfep calculations. Results of the final energy will be updated in the .csv file

The following part of the Notebook is rather time consuming, we recommend to perform those calculations on a cluster instead.

In [ ]:
```
import veloxchem as vlx
import pandas as pd

solvationfep = vlx.SolvationFepDriver()
solvationfep.padding = 1.0
filename = 'data/solvationfep_molecules.csv'

df = pd.read_csv(filename, sep=',')

results = []

for index, row in df.iterrows():

    print(f'Calculating Free Energy of Solvation for molecule: {row['SMIL
    molecule = vlx.Molecule.read_smiles(row['SMILES'])

    delta_f, final_energy = solvationfep.compute_solvation(molecule)
    results.append(f"{final_energy:.4f}")
```

```
df['Result kJ/mol'] = results
df.to_csv(filename, sep=',', index=False)
```

Plot the results for all molecules

```
In [ ]: import pandas as pd
        import matplotlib.pyplot as plt

        # Read the CSV file
        df = pd.read_csv('solvationfep_molecules.csv', sep=',')

        # Plot the results against SMILES
        plt.figure(figsize=(10, 6))
        plt.plot(df['SMILES'], df['Result kJ/mol'],'o', color='darkcyan', alpha=0

        plt.ylabel(r'$\Delta G^\mathrm{solv}$ Computed (kJ/mol)', fontsize=12)
        plt.xticks(rotation=45, ha='right', fontsize=10)
        plt.tight_layout()

        plt.show()
```

Force-Field Definition and Reparametrization

In [ ]:
```
import veloxchem as vlx
import numpy as np

# load B3LYP optimized geometry in the xyz format
molecule = vlx.Molecule.read_xyz_file(
    "data/hs276_optim_b3lyp_dev2-svp.xyz")
molecule.show(atom_indices=True, width=600, height=450)
```

This part of the code load the MM Forfield Generator and create an initial topology for the molecule object. By default, RESP charges are computed at the HF/6-31G* as recommended.

In [ ]:
```
ff_gen = vlx.MMForceFieldGenerator()
ff_gen.create_topology(molecule)
ff_gen.write_gromacs_files('HS-276_initial', 'MOL')
```

The Force-Field Generator can identify rotatable bonds.

In [ ]:
```
rot_bonds = ff_gen.rotatable_bonds
print(rot_bonds)
```

Reparametrization

We will here focus on the reparametrization of the barrier around the rotatable bond [21, 22]. We use the file 16-21-22-27.xyz from the data folder containing the results from a relaxed scan around rotatable bond [21, 22], i. e. optimized geometries and energies (in the comment line for each geometries).

In [ ]:
```
ff_gen.reparameterize_dihedrals(rotatable_bond=(21,22),
                                scan_file="data/16-21-22-27.xyz",
                                visualize=True)
```

The rotational barrier is now much improved but the relative energies between the two conformer minima are still not well reproduced, leading to significant errors in the statistical dihedral distributions. As a remedy to this situation, an additional dihedral potential can be added.

In [ ]:
```
ff_gen.add_dihedral((16, 21, 22, 27),
                    barrier=0.0,
                    phase=180.0,
                    periodicity=1)
```

We can now restart the reparametrization procedure with this added dihedral angle.

In [ ]:
```
ff_gen.reparameterize_dihedrals(rotatable_bond=(21,22),
                                scan_file="data/16-21-22-27.xyz",
                                visualize=True,
                                initial_validation=False)
```

Another dihedral angle can be added for a better agreement with the QM Potential Energy Surface.

In [ ]:
```
ff_gen.add_dihedral((16, 21, 22, 27),
                    barrier=0.0,
                    phase=0.0,
                    periodicity=4)
```

And start a new parametrization with this added dihedral angle.

In [ ]:
```
ff_gen.reparameterize_dihedrals(rotatable_bond=(21,22),
                                scan_file="data/16-21-22-27.xyz",
                                visualize=True,
                                initial_validation=False)
```

Rotatable Bond	Max difference (kJ/mol)	Std deviation (kJ/mol)
[21, 22]	0.477	0.212

With this excellent agreement between the QM and MM Potential Energy Surface, we can now save the topology as, i. e., Gromacs ouptut files.

In [9]:
```
ff_gen.write_gromacs_files('HS-276_final', 'MOL')
```

Interpolation mechanics

In [ ]:
```
import veloxchem as vlx
```

Define a VeloxChem Molecule for which the interpolation mechaics force field (IM-MM) shall be constructed.

In [ ]:
```
# Initialize the Molecule
molecule_xyz = '''16

C              1.354430000000         0.723890000000         0.062850000000
C              0.075030000000         1.193180000000        -0.131980000000
S             -1.063880000000        -0.076840000000        -0.238910000000
C              0.145050000000        -1.275960000000        -0.023130000000
C              1.392450000000        -0.699780000000         0.133940000000
C             -0.161190000000        -2.684380000000         0.016020000000
C              0.347150000000        -3.612800000000         0.907410000000
C             -0.139800000000        -4.931010000000         0.664450000000
C             -0.992970000000        -4.972470000000        -0.413030000000
S             -1.212210000000        -3.430270000000        -1.117120000000
H              2.219640000000         1.368740000000         0.151510000000
H             -0.248470000000         2.221020000000        -0.218570000000
H              2.299910000000        -1.273800000000         0.280250000000
H              1.028740000000        -3.357480000000         1.709320000000
H              0.125220000000        -5.802850000000         1.250990000000
H             -1.502580000000        -5.833680000000        -0.824080000000
'''


molecule = vlx.Molecule.from_xyz_string(molecule_xyz)
molecule.show(atom_indices=False)
```

Initialize the QM-Driver determining the underlying quality of the interpolation data.

In [ ]:
```
# Initialize the basis set and the QM driver
basis_set_label = "def2-svp"
basis = vlx.MolecularBasis.read(molecule, basis_set_label)
qm_driver = vlx.ScfRestrictedDriver()
qm_driver.xcfun = "b3lyp"
```

Define dyanmics and interpolation settings which are crucial for the quality in timing of the database. Important variables:

- .desired_point_density -> how many datapoints should the database contain for each conformer.
- .converged_cycle -> after how many cycles the construction for 1 conformer terminates (1 cycle: 3000 steps without database expansion).
- .energy_thrsh -> allowed devaition of the IM energy and QM reference calculations.

As this notebook is intended to be a practival example on how to use the IMFroceFieldGenerator but keeping the computational effort at a reasoable level, the shown settings have been used. For production run we recommend using the following settings:

- .nsteps = 50000
- .snapshots = 10000
- .desired_point_density = 80
- .converged_cycle = 5
- .energy_threshold = 2.0 kcal/mol

In [ ]:
```
# Initialize the IM force field generator with:
# dihedrals: list of dihedral angles to sample
# sampling_structures: number of structures to sample
# ensemble: NVT or NVE
# temperature: temperature of the ensemble
# nsteps: number of steps to run the dynamics
# snapshots: number of snapshots to save

imffgen_drv = vlx.IMForceFieldGenerator(qm_driver)
imffgen_drv.dihedrals_dict = [((2, 3, 5, 9), 10)]
imffgen_drv.ensemble = 'NVT'
imffgen_drv.temperature = 250
imffgen_drv.nsteps = 10000
imffgen_drv.snapshots = 5000
imffgen_drv.converged_cycle = 3
imffgen_drv.energy_threshold = 2.0
imffgen_drv.desired_point_density = 10
imffgen_drv.nstruc_to_confirm_database_quality = 10
imffgen_drv.minimize = False
```

The compute function starts the construction of a new or given database. The construction run ends by confirming the qulity of the database and the result are stored.

- note that here we provide the constructed database ('im_database.h5') as the computational cost of the second derivatives are very demanding.

In [ ]:
```
# Run the database consruction
imffgen_drv.imforcefieldfile = "data/im_database.h5"
im_results = imffgen_drv.compute(molecule, basis)
```

In [ ]:
```
ff_gen = vlx.MMForceFieldGenerator()
ff_gen.create_topology(molecule)
```

To use the constructed IM force field one has to initialize the InterpolationDriver() with user-defined settings (recommended). The current implementation is restricted to well seperated IM and MM regions and is not yet included into a linking atom framework.

In [ ]:
```
z_matrix = imffgen_drv.define_z_matrix(molecule)
im_driver = vlx.InterpolationDriver(z_matrix)
interpolation_settings = { 'interpolation_type':'shepard',
                            'exponent_p':'2',
                            'exponent_q':'2',
                            'confidence_radius':'0.5',
                            'imforcefieldfile':'im_database.h5'}
im_driver.update_settings(interpolation_settings)

openmmdyn = vlx.OpenMMDynamics()
openmmdyn.create_system_from_molecule(molecule, ff_gen, qm_atoms='all')
openmmdyn.run_qmmm(im_driver, im_driver)
```

Conformer generator

In [ ]:
```
import veloxchem as vlx
```

Systematic Search

We define a structure by using a SMILES string.

In [ ]:
```
molecule = vlx.Molecule.read_smiles(
    "CC1([C@@H](N2[C@H](S1)[C@@H](C2=O)NC(=O)CC3=CC=CC=C3)C(=O)O)C"
)  # this is SMILES for Penicillin G
molecule.show(atom_indices=True)
```

ConformerGenerator class can generate all possible conformations and apply energy minimization process with MMforcefield

In [ ]:
```
conf = vlx.ConformerGenerator()
conformers_dict = conf.generate(molecule)
```

show the lowest energy conformer

In [ ]:
```
conf.show_global_minimum()
```

show more conformers

In [ ]:
```
conf.show_conformers(number=3, atom_indices=True)
```

we can provide resp charges as partial charges to improve the used MMforcefield quality

In [ ]:
```
basis = vlx.MolecularBasis.read(molecule, "6-31g*")
resp = vlx.RespChargesDriver()
resp.ostream.mute()
partial_charges = resp.compute(molecule, basis, 'resp')
```

In [ ]:
```
conf = vlx.ConformerGenerator()
conf.partial_charges = partial_charges
conformers_dict = conf.generate(molecule)
conf.show_global_minimum(atom_indices=True)
```

we can apply implicit solvent model for the energy minimization

In [ ]:
```
conf = vlx.ConformerGenerator()
conf.ostream.mute()
conf.show_available_implicit_solvent_models()

conf.implicit_solvent_model = "obc2"
conformers_dict = conf.generate(molecule)
conf.show_global_minimum(atom_indices=True)
```

show more conformers

```
In [ ]: conf.show_conformers(number=3, atom_indices=True)
```

Extract conformers from an MD simulation

Using the OpenMMDynamics class, we can perform Molecular Dynamics

```
In [ ]: molecule = vlx.Molecule.read_xyz_file("data/tq-polymer.xyz")
        molecule.show()
```

The next cell is used to calculate RESP charges for the system. However, since this calculation is time consuming, the next cell is commented and the partial charges are provided in the following cell.

```
In [ ]: #basis = vlx.MolecularBasis.read(molecule, "6-31g*")
        #resp = vlx.RespChargesDriver()
        #partial_charges = resp.compute(molecule, basis, 'resp')
        #print(partial_charges)
```

```
In [ ]: partial_charges = [
         -0.232669,  -0.048234,  -0.007686,   0.100183,   0.386987,
         -0.249451,   0.194951,   0.114678,   0.062361,   0.005422,
         -0.107660,  -0.198262,   0.016200,  -0.090324,  -0.235291,
          0.062137,   0.419456,  -0.531796,   0.139008,   0.164115,
         -0.086069,  -0.044513,   0.199556,  -0.170296,  -0.180157,
         -0.113322,   0.158106,  -0.081966,  -0.128774,  -0.239557,
         -0.136771,   0.301582,  -0.203020,   0.138342,   0.136587,
         -0.316948,  -0.031826,   0.137154,   0.118999,   0.161926,
          0.130632,   0.173826,  -0.332761,  -0.005770,   0.079527,
          0.079527,   0.065962,   0.065962,  -0.242085,  -0.087463,
          0.012935,   0.061502,   0.266088,  -0.001114,   0.103725,
          0.180948,   0.015843,  -0.123259,  -0.242504,   0.056159,
         -0.118009,  -0.247948,   0.196117,   0.205390,  -0.298929,
          0.084877,   0.079527,   0.158266,  -0.146844,  -0.040754,
          0.251020,  -0.203210,  -0.160584,  -0.153068,   0.159255,
         -0.052933,  -0.119071,  -0.248423,  -0.079409,   0.161470,
         -0.134947,   0.149941,   0.170980,  -0.324275,  -0.042230,
          0.077237,   0.144781,   0.111871,   0.146494,   0.118468,
         -0.259568,  -0.020448,   0.079707,   0.079707,   0.065165,
          0.065165,   0.065962,   0.065165,   0.079707,   0.091273,
         -0.257720,  -0.061333,   0.006290,   0.053719,   0.241539,
          0.008275,   0.177544,   0.136687,   0.102227,   0.036805,
         -0.167956,  -0.170007,   0.026697,  -0.121728,  -0.219001,
          0.179470,   0.218907,  -0.269483,   0.130723,   0.169499,
         -0.141246,  -0.045503,   0.231592,  -0.203064,  -0.149092,
         -0.160352,   0.156878,  -0.087277,  -0.117300,  -0.253518,
         -0.105665,   0.201677,  -0.129198,   0.154380,   0.144414,
         -0.311560,  -0.050335,   0.169505,   0.117806,   0.078577,
          0.104835,   0.177924,  -0.277724,  -0.030888,   0.083872,
          0.083872,   0.071197,   0.071197,  -0.205491,  -0.105325,
          0.004215,   0.071387,   0.205212,   0.002548,   0.100560,
          0.158667,   0.021198,  -0.109115,  -0.258546,   0.071271,
         -0.137911,  -0.231297,   0.150851,   0.232605,  -0.255901,
          0.069410,   0.083872,   0.165216,  -0.118096,  -0.035060,
          0.232363,  -0.199482,  -0.160185,  -0.163606,   0.159908,
```

```
 -0.075661,  -0.119060,  -0.255872,  -0.099171,   0.211171,
 -0.146255,   0.167897,   0.176623,  -0.312825,  -0.059878,
  0.072443,   0.144852,   0.119482,   0.152389,   0.115928,
 -0.288919,  -0.023122,   0.084877,   0.084877,   0.069835,
  0.069835,   0.071197,   0.069835,  -0.277164,  -0.038921,
  0.002052,   0.076138,   0.220403,   0.008773,   0.186555,
  0.122042,   0.064124,   0.006807,  -0.102455,  -0.211713,
  0.025525,  -0.100865,  -0.225711,   0.134458,   0.220855,
 -0.239842,   0.146481,   0.157469,  -0.084636,  -0.038788,
  0.191114,  -0.167495,  -0.173886,  -0.139361,   0.158124,
 -0.115619,  -0.110832,  -0.246506,  -0.111697,   0.200255,
 -0.102728,   0.147022,   0.137019,  -0.311401,  -0.028076,
  0.157916,   0.121854,   0.070941,   0.097209,   0.174835,
 -0.277731,  -0.058498,   0.078470,   0.078470,   0.079650,
  0.079650,  -0.239752,  -0.093417,   0.013395,   0.065722,
  0.252234,   0.002507,   0.097142,   0.176412,   0.012295,
 -0.109919,  -0.253222,   0.062514,  -0.121338,  -0.239217,
  0.179990,   0.225267,  -0.293594,   0.084407,   0.078470,
  0.163374,  -0.156555,  -0.027891,   0.267086,  -0.220163,
 -0.153943,  -0.138332,   0.156524,  -0.068274,  -0.107757,
 -0.248650,  -0.083734,   0.150618,  -0.128876,   0.155916,
  0.171802,  -0.332583,  -0.033320,   0.071492,   0.147678,
  0.114573,   0.140265,   0.115307,  -0.249021,  -0.028279,
  0.077074,   0.077074,   0.068191,   0.068191,   0.079650,
  0.068191,   0.077074,   0.085617,  -0.262181,  -0.058425,
  0.004567,   0.054389,   0.245865,   0.006966,   0.178357,
  0.134007,   0.098000,   0.031254,  -0.152193,  -0.172248,
  0.024457,  -0.114391,  -0.218354,   0.183237,   0.211562,
 -0.273990,   0.125284,   0.158656,  -0.144065,  -0.033540,
  0.216218,  -0.188581,  -0.155284,  -0.157807,   0.156199,
 -0.078857,  -0.117411,  -0.254610,  -0.097991,   0.198924,
 -0.133192,   0.153092,   0.142005,  -0.306650,  -0.071477,
  0.168597,   0.113979,   0.075060,   0.107961,   0.177382,
 -0.281830,  -0.022927,   0.092650,   0.092650,   0.069410,
  0.069410,  -0.234824,  -0.088279,  -0.010610,   0.098104,
  0.235745,  -0.003237,   0.091918,   0.181039,  -0.033203,
 -0.007199,  -0.314039,  -0.121585,  -0.064129,  -0.269034,
  0.181444,   0.222313,  -0.282548,   0.200030,   0.092650,
  0.175556,  -0.124800,  -0.033037,   0.242958,  -0.205747,
 -0.157096,  -0.151879,   0.158719,  -0.061573,  -0.112321,
 -0.250936,  -0.103306,   0.194760,  -0.137261,   0.151524,
  0.175522,  -0.320888,  -0.048925,   0.069816,   0.146477,
  0.121450,   0.141797,   0.115735,  -0.282016,  -0.026049,
  0.084407,   0.084407,   0.070941,   0.070941]
```

In the next cell, we are using the partial charges to derive a Force-Field using the MMForceFieldGenerator class.

In [ ]:
```
ff_gen = vlx.MMForceFieldGenerator()
ff_gen.partial_charges = partial_charges
ff_gen.create_topology(molecule)
```

Once a force field is derived, different functions can be used which use the OpenMM library to perform molecular dynamics simulations. This is available in the OpenMMDynamics class. The next cell gives an example how to run a MD.

In [ ]:
```
opm_dyn = vlx.OpenMMDynamics()
opm_dyn.create_system_from_molecule(molecule,
```

```
                                      ff_gen,
                                      filename='tq-polymer',
                                      residue_name='MOL')
opm_dyn.run_md(
            ensemble='NVE',
            temperature=300,
            timestep=2.0,
            nsteps=100000,
            traj_file='tq_polymer_md.pdb')
```

Moreover, we have developed a function to extract conformers from the MD simulation at high temperature. The user can define a number of snapshots that will be optimized and save. In addition, those snapshots can be filtered to avoid that the same conformer is present multiple timess by using the option unique_conformers.

In [ ]:
```
conformers_dict = opm_dyn.conformational_sampling(
                                ensemble='NVT',
                                temperature=1000,
                                timestep=2.0,
                                nsteps=1000000,
                                snapshots=5000,
                                unique_conformers=True,
                                qm_driver=None,
                                basis=None,
                                constraints=None)
```

In [ ]:
```
opm_dyn.show_conformers(number=10)
```

In [ ]:
```
# plot the distribution of the relative energies of the conformers
import matplotlib.pyplot as plt
import numpy as np

fig, ax = plt.subplots(figsize=(8, 4))
ax.hist(conformers_dict['energies']-np.min(conformers_dict['energies']),
        bins=50,
        color='darkcyan',
        alpha=0.7)
plt.xlabel('Relative Energy (kJ/mol)')
plt.ylabel('Number of Conformers')

plt.savefig('conformer_energy_distribution.pdf')
plt.show()
```

In [ ]:
```
# show the most stable conformer and write it to an XYZ file
conformers_dict['molecules'][0].show()
conformers_dict['molecules'][0].write_xyz_file('tq-polymer-0.xyz')
```

EVB

In [ ]:
```
import veloxchem as vlx
```

Create the driver object for the EVB protocol, build the forcefields from two xyz files supplied in the `data` folder and then build the OpenMM systems that will be used for the FEP.

In [ ]:
```
CH3Cl_Br = vlx.Molecule.read_xyz_file('data/CH3Cl_Br.xyz')
CH3Cl_Br.set_charge(-1)
CH3Br_Cl = vlx.Molecule.read_xyz_file('data/CH3Br_Cl.xyz')
CH3Br_Cl.set_charge(-1)

evb = vlx.EvbDriver()
evb.name = 'Sn2'
evb.build_ff_from_molecules([CH3Cl_Br],
                            [CH3Br_Cl])

evb.build_systems(['vacuum', 'water', 'dmf', 'acetone'])
```

Run the FEP and compute the free energy profiles based on reference values. These values correspond to the first environment specified in the `build_systems` function, which in this case is the vacuum configuration. This will take a significant amount of time to run. It is recommended to use GPU acceleration for this. See http://docs.openmm.org/latest/userguide/application/01_getting_started.html.

In [ ]:
```
evb.run_FEP()
results = evb.compute_energy_profiles(barrier=42, free_energy=13.5)
```

The results can then plotted

In [ ]:
```
evb.plot_results(results)
```
